# Homogeneous fluorescent specific PCR for the authentication of medicinal snakes using cationic conjugated polymers

**DOI:** 10.1038/srep16260

**Published:** 2015-11-05

**Authors:** Chao Jiang, Yuan Yuan, Libing Liu, Jingyi Hou, Yan Jin, Luqi Huang

**Affiliations:** 1 State Key Laboratory Breeding Base of Dao-di Herbs, National Resources Center for Chinese Materia Medica, China Academy of Chinese Medical Sciences, Beijing, 100700, P.R. China; 2 Beijing Area Major Laboratory of Protection and Utilization of Traditional Chinese Medicine, College of Resources, Beijing Normal University, Beijing, 100875, P.R. China; 3 Beijing National Laboratory for Molecular Sciences, Key Laboratory of Organic Solids, Institute of Chemistry, Chinese Academy of Sciences, Beijing 100190, P. R. China

## Abstract

A label-free, homogenous and sensitive one-step method for the molecular authentication of medicinal snakes has been developed by combining a rapid PCR technique with water-soluble cationic conjugated polyelectrolytes (CCPs). Three medicinal snake materials (*Deinagkistrodon acutus*, *Zaocys dhumnades* and *Bungarus multicinctus*; a total of 35 specimens) and 48 snake specimens with similar morphologies and textures were clearly distinguished by the naked eye by utilizing a CCP-based assay in a high-throughput manner. The identification of medicinal snakes in patented Chinese drugs was successfully performed using this detection system. In contrast to previous fluorescence-labeled oligonucleotide detection and direct DNA stain hybridization assays, this method does not require designing dye-labeled primers, and unfavorable dimer fluorescence is avoided in this homogenous method.

Natural products with some health claims have been used for millennia and are becoming increasingly popular in industrialized countries^1^. Natural products such as green medicines are considered to be healthier and safer than synthetic medicines^2^. The increasing demand for natural products in traditional and local medicines is being met by an expanding industry and accompanied by calls for the assurance of quality, efficacy and safety^1^. The substitution or adulteration of authentic crude drugs is motivated by the desire for financial gain or a lack of taxonomical knowledge. Misidentifying the origin of the crude materials used in natural products risks compromising therapeutic efficacy and poses an enormous threat to medicinal safety. Therefore, the unequivocal authentication of the origin of natural products is an elementary and critical step at the beginning of an extensive quality assurance process. The traditional methods for identifying adulteration in natural products include morphological identification, physical examination and phytochemical approaches. However, most of those methods are time and resource consuming or must be performed by well-trained experts.

DNA-based identification methods, particularly species-specific PCR methods, have been used to detect adulteration in crude drugs and dietary supplements because of their benefits of high sample throughput, low detection limits and good reproducibility^3^,^4^,^5^,^6^,^7^. Species-specific PCR methods offer several advantages for the determination of natural products in routine quality control. Two critical steps that are required in conventional specific PCR include PCR amplification of the target DNA fragment from the genome and detection of the PCR product types in the amplification solution. Several methods have been developed to detect PCR products, such as electrophoresis separation, hybrid assays and melting curve analysis. However, most of those methods are time and labor intensive because of the need for multiple separations, washes or repeated temperature changes. Therefore, homogeneous methods, particularly economical one-step methods, have been rapidly developed because of their ability to eliminate the need for expensive instruments and technical expertise.

Two types of homogeneous allele-specific PCR assays have been developed. The first is fluorescence-labeled oligonucleotide detection, e.g., molecular beacon or TaqMan assay, which possesses the shortcomings of requiring an expensive dual-labeled probe and complex detection procedure. The second is direct DNA stain hybridization assays. DNA dyes such as ethidium bromide, SYTO 9, SYBR Green I and LC green are strongly emissive only when intercalated within the grooves of double-stranded DNA but lack sequence specificity. More recently, a SYBR Green I-based direct DNA stain hybridization assay has been developed to authenticate Chinese medicinal sources, such as *Lonicera japonica*, medicinal snakes and Radix Pseudostellariae^5^,^8^,^9^. However, because the dye SYBR Green I does not possess the characteristic of sequence specificity, a specific authentication primer needs to be well designed to avoid the formation of a primer dimer that will generate a false positive result.

Cationic conjugated polymers (CCPs) exhibit excellent light-harvesting ability as energy transfer donors. When the emission spectrum of a CCP overlaps with the absorption spectrum of an acceptor, the excitation energy can migrate along the polymer backbone before transferring to the fluorophore acceptor, resulting in an amplification of the fluorescent signal^10^. Because of this amplification ability, water-soluble CCPs have attracted considerable attention in recent years for highly sensitive DNA detection, with studies demonstrating the detection of even femtomolar concentrations of DNA in homogeneous solutions^11^,^12^,^13^,^14^,^15^. Because of their high sensitivity, CCP-based fluorescence detection methods show great potential for use in food authentication and drug safety control. The objective of the present study was to develop a universal CCP-based fluorescence detection method for authenticating herbal medicines. Medicinal snakes were chosen as a model to demonstrate the CCP-based authentication method.

The medicinal snakes QS (*Deinagkistrodon acutus*), WSS (*Zaocys dhumnades*) and JQ (*Bungarus multicinctus*) have been officially recorded in the Chinese Pharmacopoeia since 1953. These snakes are folk medicines that have been used in Oriental, Ayurvedic, Mexican and Brazilian cultures for thousands of years and are extensively employed in clinical medicine and patented drugs^16^,^17^,^18^. Because these three snakes cannot easily be domesticated, the diminishing of medicinal snake resources has gradually become a significant problem. *D. acutus* was even listed in the Convention on International Trade in Endangered Species of Wild Fauna and Flora (CITES)^19^. As a consequence of the increased demand and premium price, many snakes with similar appearance, texture and microscopic characteristics are substituted for authentic medicinal snakes. Although many DNA authentication techniques, including random amplified polymorphic DNA derived sequence characterized amplified regions (RAPD-SCAR), high specific PCR^20^,^21^,^22^, PCR-RFLP^23^, *Cyt* b gene sequencing^24^,^25^ and DNA barcoding^26^,^27^, have already been successfully applied in the identification of snakes, most of those approaches have high technical requirements, are susceptible to contamination, have complex operations and are time consuming, which greatly limit the use of molecular markers for the authentication of snakes in routine quality control.

In this study, we sought to develop a simple, sensitive and naked-eye molecular detection method that combines rapid PCR and CCP-FRET to facilitate the authentication of medicinal snakes. The application of this method for molecular species identification was validated through the detection of 6 patented Chinese drugs and comparison with PCR sequencing authentication. Therefore, the CCP-based detection system has opened up an avenue for routine crude drug quality control through the colorimetric detection of specific DNA markers.

## Results

### Principles of authentication

**Figure 1:**
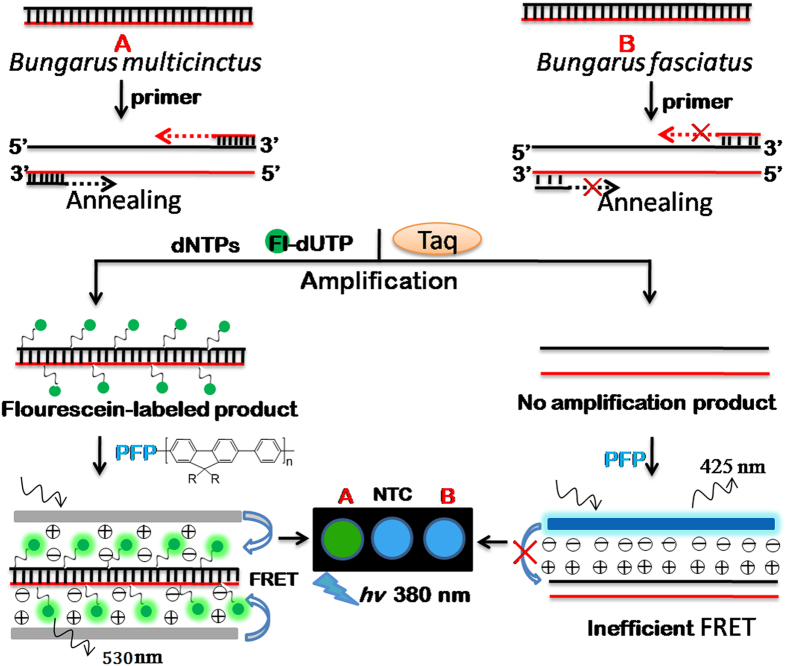
Schematic principle of the conjugated polymer-based assay method for the authentication of medicinal snakes.

Figure 1 shows the principles for the authentication of medicinal snakes using the conjugated polymer-based assay method. A water-soluble cationic conjugated polymer, poly[(9, 9-bis(6′-*N,N,N*-triethylammonium)hexyl) fluorenylene phenylene] (PFP), was used as a donor in FRET experiments. Fluorescein-labeled dUTP (Fl-dUTP) was selected as an acceptor. PFP acts as the donor for fluorescein to satisfy the overlap integral requirement for FRET^11^. In situation A, with the authentic *B. multicinctus* DNA containing the target template fragment and the *B. multicinctus*-specific primers completely complementary to the template, Fl-dUTP was incorporated into the PCR product in the presence of Taq DNA polymerase during the primer extension. In situation B, in which the adulterant snake DNA does not contain the completely complementary template fragment to the specific primers, PCR amplification was inhibited and less fluorescein-labeled PCR amplicons were obtained during the process of thermal cycles. Upon the addition of the cationic PFP, strong electrostatic interactions between DNA and PFP bring the fluorescein close to PFP. Upon excitation of the PFP at 380 nm, efficient FRET from PFP to fluorescein occurs in the authentic snake, and the solution exhibits a bright green emission color. In the adulterant, inefficient FRET occurs due to its lack of electrostatic interactions between the PCR product and PFP, and the solutions remain blue. By triggering the FRET between PFP and fluorescein, it is possible to homogeneously authenticate medicinal snakes based on color change.

### CCP-based assay method for authentication medicinal snake

**Figure 2:**
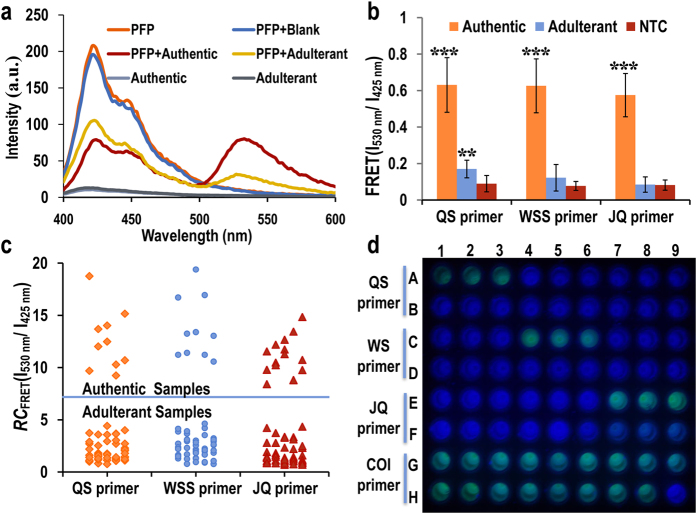
(**a**) Emission spectra from solutions containing PFP, *B. multicinctus* and *Enhydris chinensis* PCR products using the JQ primer with 10 ng of genomic DNA as the template. The excitation wavelength is 380 nm. (**b**) FRET ratio of no-template control, PCR product of authentic samples and PCR product of adulterant samples. The two-tail T test: **p < 0.01, ***p < 0.001 compared with the blank control. For the mean, error bars indicate ± S.D. (**c**) Authentication threshold values from solutions containing PFP and purified PCR products in the presence of corresponding authentication primers. (**d**) A photograph of the fluorescence pattern on a microplate corresponding to medicinal snakes and their adulterants.

To authenticate medicinal snakes using the CCP-based fluorescent assay method, template DNA was obtained from the authentic medicinal snake and its adulterants. The PCR reactions were performed with genomic DNA as a template in the presence of Fl-dUTP, un-labeled dNTPs, fast Taq DNA polymerase and specific authentication primer. As demonstrated in Fig. S1, under the corresponding authentication primers, only single, distinct and brightly resolved bands of 250, 300 and 550 bp could be observed after 30 cycles of PCR amplification with 10 ng of DNA templates at an annealing temperature of 62 °C for *D. acutus, Z. dhumnades* and *B. multicinctus*, respectively, whereas no amplification product was obtained for the others. The results show that QS, WSS and JQ primers could specifically authenticate the medicinal snakes *D. acutus, Z. dhumnades* and *B. multicinctus*. Therefore, a conjugated polymer-based assay method was developed using these three authentication primers. Images of the amplification products with 20 μL of PFP dropped on a 96-well PCR plate under 380 nm UV light in transmission mode are presented in Fig. 2d. Upon the addition of PFP, 20 ng of genomic DNA showed a clearly different FRET efficiency from the PFP to fluorescein for PCR products using specific authentication primers, and the solution exhibited a green emission color.

Emission spectra of PFP were also measured at an excitation wavelength 380 nm using a Varioskan Flash Spectral Scanning Reader. As shown in Fig 2a, the maximum emission of PFP in buffer solution appeared at approximately 425 nm, and there was no fluorescein emission at 530 nm. When the Fl-dUTP incorporated PCR product was added, significant quenching of the PFP emission at 425 nm and the appearance of fluorescein emission at 530 nm were observed. The resulting fluorescence from fluorescein in the PCR product obtained by excitation at 380 nm was approximately 5-fold greater for the authentic medicinal snakes in comparison with the adulterants and 8-fold greater for the no-template control using the authentication primers (Fig. 2b). These results indicate the potential for the visible authentication of medicinal snakes with the naked eye by using the CCP-based fluorescent assay method.

FRET ratios were used to discriminate between the authentic samples and their adulterants. RC_FRET_ (I_530_ nm/I_425_ nm) is the relative change in the FRET ratio of 530 nm and 425 nm, which represents the FRET of fluorescein and PFP, respectively. Threshold values of *D. acutus, Z. dhumnades* and *B. multicinctus* were determined as RC_FRET_ (I_530_ nm/I_425_ nm) > 4.5, 4.7, and 4.3 using the corresponding authentication primers, respectively (Fig. 2c). Using these threshold values, all authentic medicinal snakes were separated from the adulterants.

### Methodological studies

**Figure 3:**
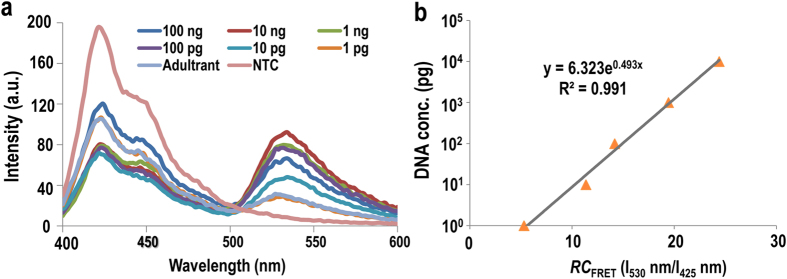
(**a**) Emission spectra from solutions containing PFP and PCR products using the JQ primer in the presence of varying concentrations of *B. multicinctus* genomic DNA. No-template control (NTC) was used as the blank. (**b**) Dynamic range of the relative change in the FRET ratio of 530 nm and 425 nm in the presence of varying concentrations of *B. multicinctus* genomic DNA.

Figure 3a compares the emission spectra observed upon adding PFP ([PFP] = 2.0 × 10^−7^ M in monomer repeat units (RUs)) to PCR products using *B. multicinctus*-specific primer with the authentic *B. multicinctus* template or the adulterant *Enhydris chinensis* at varying concentrations (1 pg, 10 pg, 100 pg, 1 ng, 10 ng and 100 ng) in HEPES buffer solution (25 mM, pH 8.0). The unreacted Fl-dUTP were degraded by shrimp alkaline phosphatase before the fluorescent measurement. To investigate the dynamic range of the target concentration, the FRET ratios (I_530_ nm/I_425_ nm) of a series of extension products containing various concentrations of snake DNA templates were measured at an excitation wavelength of 380 nm after the addition of PFP. As shown in Fig. 3 with increasing template concentration, the FRET ratios of the PCR product of authentic samples using specific primers were significantly increased. Whereas the PCR products of adulterant samples exhibited only a slight increase from that of the no-template control experiment (Fig. 2b). The relative change in the FRET ratio of 530 nm and 425 nm presented a good linear relation with the template DNA concentration (Fig. 3b). The dynamic ranges at which the DNA separates from *D. acutus*, *Z. dhumnades* and *B. multicinctus* were 10 pg to 10 ng, 1 pg to 10 ng and 10 pg to 10 ng in a 20 μL PCR reaction system, respectively. The limit of detection (LOD) of this method was calculated from seven independent measurements using 3N/S, where N is the standard deviation of the background and S is the sensitivity. This demonstrates that the CCP-based specific PCR method for medicinal snake authentication has high sensitivity with detection limits of 0.27 pg, 0.21 pg and 0.18 pg for *D. acutus*, *Z. dhumnades* and *B. multicinctus*, respectively.

### Patented Chinese drugs sample identification

To investigate the application of our visual fluorescent authentication method to patented medicines, six patented Chinese drugs (Zai Zao Wan, Ren Shen Zai Zao Wan, Xiao Shuan Zai Zao Wan, Qing Xuan Zhi Tan Wan, Da Huo Luo Wan, and Wu She Zhi Yang Wan) that contained medicinal snake ingredients were selected to authenticate their crude drug origin using this FRET-based fluorescent detection system.

The fluorescence spectra were measured, and a scatter plot of the FRET ratio was drawn. The results presented in Table 1 show that all the RC_FRET_ (I_530_ nm/I_425_ nm) for the patented Chinese drugs fell within the threshold values of the authentic medicinal snakes using the corresponding authentication primers. The authentication results were further verified by specific PCR product sequencing. Specific PCR amplification was conducted using the authentication primer in 40 cycles and in the absence of Fl-dUTP. Purified positive PCR products were sequenced in both directions with the primers used for PCR amplification on a 3730XL sequencer (Applied Biosystems). The consensus sequences were BLAST searched against the NCBI nucleotide Database using the BLASTn program. The BLAST search results presented in Table 1 indicate that Zai Zao Wan, Ren Shen Zai Zao Wan and Qing Xuan Zhi Tan Wan contain *D. acutus*; Wu She Zhi Yang Wan contains *Z. dhumnades*; Xiao Shuan Zai Zao Wan contains *B. multicinctus;* and Da Huo Luo Wan contains both *D. acutus* and *Z. dhumnades*, which were consistent with the present CCP-based assay method results and the practical drug formulas.

## Discussion

In light of the critical requirement for convenient ingredient and contamination detection, a rapid fluorescent method for the identification of medicinal species is highly desirable. Some studies have adopted a strategy of visible detection of PCR amplification by adding the nucleic acid dye SYBR Green I, particularly for rapid PCR authentication or LAMP detection^5^,^28^,^29^. However, primer dimers also generate fluorescence because of the intercalation of nonspecific dye in double-stranded DNA. Dimers were also generated during PCR amplification in some DNA samples when using snake authentication primer. Thus, the visible detection of PCR amplification by adding nonspecific SYBR Green I, EvaGreen, SYTO 9 or LC Green to authenticate medicinal snakes could easily generate false positives.

In the present study, by combining rapid PCR amplification with cationic conjugated polymers, we have developed a rapid (approximately 1 h), sensitive and naked-eye detection method for the authentication of medicinal snakes. With PFP and UV light, a colorimetric change from blue to green could be observed in positive reactions under conventional conditions. Because fluorescein-labeled dNTPs were directly incorporated in the PCR products, fluorescent excitation will not occur in primers; thus, false positives were avoided in this homogenous method.

PFP possesses an excellent optical amplification property. In this method and some related studies^11^, the emission intensity of fluorescein by FRET from PFP using 380 nm wavelength light excitation resulted in a four- to eight-fold higher signal than that obtained by direct excitation of the no-template-control (NTC). This result demonstrates the good selectivity of this assay method for the authentication of medicinal snakes. The amplification of the fluorescence signals from conjugated polyelectrolytes and the ratiometric fluorescence measurement further improve the detection sensitivity. Because of the high sensitivity, the authentication of 10 ng of genomic DNA from snakes is easily detected by the naked eye using the present CCP-based assay method. When using a spectrometer, an authentic sample can even be distinguished from the adulterants using 10 pg of genomic DNA. Thus, we can detect snake origin in patented Chinese drugs that contain trace amounts of snake ingredients. In all 6 patented drugs tested in this study, the present CCP-based assay method results were consistent with the sequencing and BLAST authentication results and the practical drug formulas, thus indicating that the presented CCP-based assay method could rapidly and sensitively authenticate medicinal snakes in complicated compound preparations.

## Methods

### Specific PCR assays

PCR amplification was performed in a total volume of 20 μL containing 2 μL of 10 × Fast Buffer I (Takara, China), 1 μL of dNTP mixture (100 μM each, Takara), 0.2 μL of SpeedStar HS *Taq* DNA polymerase (5 U/μL, Takara), 2 μL of Fl-dUTP (25 nM, Perkin Elmer), 0.2 μL of identification primer (10 μM, Sangon), 0.5 μL of 10 mg/mL bovine serum albumin (Sigma-Aldrich, St. Louis, MO, USA), 1 μL of 20% Polyvinyl pyrrolidone 40 (Sigma-Aldrich, St. Louis, MO, USA) and a total of 10 ng of DNA. Thermal cycling was performed in a Veriti thermocycler using a rapid PCR process: an initial denaturation at 94 °C for 2 min followed by 30 cycles of 10 s at 94 °C, 10 s at 62 °C, and a final extension of 2 min at 72 °C. After the reaction cycle, the reaction products were held at 4 °C.

### Electrophoresis analysis

After the reaction, 5 μL of PCR reaction product was analyzed on 2% agarose gels using TAE buffer (40 mM Tris/acetate, 2 mM EDTA, pH 8.0) at 120 V (PowerPac 1000, Bio-Rad Laboratories Inc., Hercules, CA, USA). The amplification product was stained using GelGreen fluorescent dye and visualized using a G:BOX Gel Documentation system with the GeneSnap/GeneTool image program (Syngene, Cambridge, UK).

### Fluorescence detection

Prior to the fluorescence measurement, 2.5 μL of 10 × SAP buffer (NEB, China) and 2 μL of shrimp alkaline phosphatase (0.5 U/μL) were added to the PCR products and incubated at 37 °C for 20 min to degrade the unreacted Fl-dNTP. Following incubation, the mixture was held at 4 °C. For the fluorescence detection based on the fluorescence resonance energy transfer (FRET) ratio, 80 μL of 25 mM HEPES buffer, 20 μL of 15 μM PFP and 20 μL of PCR products were added to 96-well microtiter plates (Thermo Scientific). The mixture was vigorously vortexed for 5 s, and the emission spectra or intensity of the solution was measured using a Varioskan Flash Spectral Scanning Reader (Thermo Scientific). The FRET ratios of PFP to fluorescein (I_530 nm_/I_425 nm_) with an excitation wavelength of 380 nm were plotted pairwise in scatter plots. For the visible detection of FRET, 20 μL of PFP was added to the 96-well PCR plate containing PCR products and mixed thoroughly by pipetting. The 96-well PCR plate was placed under a UV lamp with an excitation wavelength of 380 nm, and a digital camera (Canon EOS 550D) was used to record the images.

### Controls

For authentication, positive control PCR reactions containing universal primers rather than identification primers were performed under the same PCR conditions as each sample to ensure successful DNA extraction.

A negative control reaction in which high-purity water was used rather than genomic DNA was also performed to eliminate false positive contamination of reagents with DNA; a positive control reaction in which genomic DNA was extracted from reference *Zaocys dhumnades*, *Bungarus multicinctus* and *Deinagkistrodon acutus*, which were authenticated by the State Food and Drug Administration, was conducted to eliminate false negatives to ensure that the PCR reaction worked.

## Additional Information

**How to cite this article**: Jiang, C. *et al.* Homogeneous fluorescent specific PCR for the authentication of medicinal snakes using cationic conjugated polymers. *Sci. Rep.* **5**, 16260; doi: 10.1038/srep16260 (2015).

## Supplementary Material

Supplementary Information

## Figures and Tables

**Table 1:** Relative change in the FRET ratio and the BLAST results in patented Chinese drugs.

Chinese patent medicine	RC_FRET_ (I_530_ nm/I_425_ nm)			BLAST result (Best Hit)			
	QS primer	WSS primer	JQ primer	Species	E value	Identity (%)	Accession
Zai Zao Wan	12.67	4.20	3.34	*D. acutus*	1e-141	99	AF038883.1
Ren Shen Zai Zao Wan	10.23	3.01	2.34	*D. acutus*	7e-149	99	AF038883.1
Qing Xuan Zhi Tan Wan	9.79	1.68	2.79	*D. acutus*	7e-148	98	EU913476.1
Da Huo Luo Wan	7.85	8.48	4.23	*D. acutus*^a^ *Z. dhumnades*^b^	3e-147^a^ 1e-116^b^	98^a^ 96^b^	AY223560.1^a^ AF236676.2^b^
Wu She Zhi Yang Wan	4.20	12.02	2.48	*Z. dhumnades*	1e-110	95	AF236676.2
Xiao Shuan Zai Zao Wan	3.01	3.66	9.68	*B. multicinctus*	0	99	AJ749344.1

^a^PCR amplification using the QS primer.

^b^PCR amplification using the WSS primer.
