# Supplementary material for: Homogeneous fluorescent specific PCR for the authentication of medicinal snakes using cationic conjugated polymers
Source: Sci Rep. 2015 Nov 5;5:16260. doi: 10.1038/srep16260 (PMC4633673; doi:10.1038/srep16260)
Supplement: Supplementary Information [file srep16260-s1.doc]

**Supplementary materials**

**Homogeneous fluorescent specific PCR for the authentication of medicinal snakes using cationic conjugated polymers**

Chao Jiang^1,2^, Yuan Yuan^1,*^, Libing Liu^3^, Jingyi Hou^2^, Yan Jin^1^ & Luqi Huang^1,*^

^1^State Key Laboratory Breeding Base of Dao-di Herbs, National resource center for Chinese Materia Medica, China Academy of Chinese Medical Sciences, Beijing, 100700, P.R. China

^2^Beijing Area Major Laboratory of Protection and Utilization of Traditional Chinese Medicine, College of resources, Beijing Normal University, Beijing, 100875, P.R. China

^3^Beijing National Laboratory for Molecular Sciences, Key Laboratory of Organic Solids, Institute of Chemistry, Chinese Academy of Sciences, Beijing 100190, P. R. China

***Address for correspondence**

Dr. Yuan Yuan and Prof. Dr. Luqi Huang

State Key Laboratory Breeding Base of Dao-di Herbs, National resource center for Chinese Materia Medica, China Academy of Chinese Medical Sciences, Beijing, 100700, P.R. China

[huangluqi01@126.com](mailto:huangluqi01@126.com) (LQ Huang); [yyuan0732@gmail.com](mailto:yyuan0732@gmail.com) (Y Yuan);

Tel: +86 10 64014411-2851

Fax: +86 10 64013996

**Materials and measurements** PAGE-purified primers were synthesized by Sangon Biotech Co., Ltd (Shanghai, China). SpeedStar HS Taq DNA polymerase was obtained from Takara Biotech Co., Ltd. (Dalian, China). Shrimp alkaline phosphatase (SAP) was obtained from New England Biolabs (Beijing, China). Fl-dUTP was purchased from Perkin Elmer (Massachusetts, USA). GelGreen dye was obtained from Tiangen Biotech Co., Ltd. (Beijing, China). The synthesis of PFP was performed as described in the literature^1^. PCR reactions were performed in a Veriti® thermocycler (Applied Biosystems, Foster City, CA, USA). An F-4500 spectrofluorometer (Hitachi, Ltd, Tokyo, Japan) equipped with a 150 W high-pressure Xenon lamp was used to obtain the fluorescence spectra. The image was recorded using a Pentax K-7 digital camera in a WD-9403F UV Viewing Cabinet (Beijing Liuyi Instrument Factory, Beijing) equipped with a 550–650 nm band-pass filter.

**Samples and DNA isolation** Information on the tested samples is listed in Table S1. Fifteen specimens of dried *Bungarus multicinctus* snakelet, ten specimens of *Zaocys dhumnades*, Ten specimens of *Deinagkistrodon acutus* and forty-eight specimens of commercially available adulteration samples belonging to 17 genera, such as *Daboia russelii*, *Bungarus fasciatus*, and *Ptyas mucosus* etc.*,* were collected from herbal markets. All of the collected samples were identified by a taxonomist based on their morphological characteristics. Their identities were further confirmed by conventional PCR and sequencing.

The snake materials were frozen in liquid nitrogen and ground to a fine powder using a MM 400 Mixer Mill (Retsch Technology GmbH, Haan, Germany). At least 1 g of snake material was powdered, and approximately 25 mg of powder was randomly selected for DNA extraction. DNA was extracted using the DNeasy Blood & Tissue Kit (QIAGEN, Valencia, CA) following the manufacturer’s instructions and stored at -20 ℃. The concentration of the isolated DNA and the ratio of absorbances at 260 nm to 280 nm (OD_260_/OD_280_ ratio) were measured using a NanoDrop ND-1000 spectrophotometer (Gene, Hong Kong, China). The snake DNA was eluted with double-distilled sterile water to 10 ng/μL for the PCR reaction.

Six patented Chinese drugs that were purchased from a local pharmacy in Beijing, P.R. China, were also tested in this study (details in Table S2). To protect the manufacturers’ identities, the sample sources are described by their preparation names. Approximately 100 mg of patented drug powders were randomly selected for DNA extraction. DNA was extracted using the modified CTAB method^2^ and purified using a GeneJET Gel Extraction Kit (Thermo Scientific) following the standard protocol.

Table S1 Detailed information of snake materials in this paper

| **No.** | **Species** | **Numbers** | **Collected area** |
| --- | --- | --- | --- |
| **1** | *Zaocys dhumnades* | 10 | Bozhou, China |
| **2** | *Bungarus multicinctus* | 15 | Bozhou, China |
| **3** | *B. fasciatus* | 3 | Bozhou, China |
| **4** | *Deinagkistrodon acutus* | 10 | Yulin, China |
| **5** | *Naja naja* | 3 | Bozhou, China |
| **6** | *Enhydris chinensis* | 3 | Bozhou, China |
| **7** | *Enhydris plumbea* | 3 | Bozhou, China |
| **8** | *Lycodon rufozonatus* | 3 | Guangzhou, China |
| **9** | *Agkistrodon halys* | 2 | Bozhou, China |
| **10** | *Oocatochus rufodsata* | 3 | Guangzhou, China |
| **11** | *Ptyas korros* | 2 | Bozhou, China |
| **12** | *P. mucosus* | 3 | Bozhou, China |
| **13** | *Gloydius brevicaudus* | 1 | Bozhou, China |
| **14** | *Elaphe carinata* | 3 | Bozhou, China |
| **15** | *E. taeniura* | 3 | Bozhou, China |
| **16** | *E. radiata* | 2 | Anguo, China |
| **17** | *Pelamis platurus* | 2 | Bozhou, China |
| **18** | *Orthriophis moellendorffi* | 2 | Anguo, China |
| **19** | *Sinonatrix annularis* | 3 | Anguo, China |
| **20** | *Rhabdophis tigrina* | 2 | Chengdu, China |
| **21** | *Dinodon rufozonatum* | 3 | Bozhou, China |
| **22** | *Xenochrophis flavipunctatus* | 1 | Guangzhou, China |
| **23** | *Lycodon ruhstrati* | 1 | Guangzhou, China |

Table S2 Patented Chinese drug samples used in this study

| Chinese  patent medicine | Raw materials number | Adjuvant  material | Contain |
| --- | --- | --- | --- |
| Zai Zao Wan | 58 | honey | *D. acutus* |
| Ren Shen Zai Zao Wan | 56 | honey | *D. acutus* |
| Xiao Shuan Zai Zao Wan | 38 | honey | *B. multicinctus* |
| Qing Xuan Zhi Tan Wan | 39 | honey | *D. acutus* |
| Da Huo Luo Wan | 50 | honey | *D. acutus & Z. dhumnades* |
| Wu She Zhi Yang Wan | 11 | talcum powder,  red ferric oxide | *Z. dhumnades* |

Table S3 Primer sequences in this paper

| **Identification Primer Sets (5’→3’)** |
| --- |
| Forward and Reverse primer for *Z. dhumnades* (WSS primer) |
| GCGAAAGCTCGACCTAGCAAGGGGACCACA  CAGGCTCCTCTAGGTTGTTATGGGGTACCG |
| Forward and Reverse primer for *B. multicinctus* (JQ primer) |
| GAAATTTCGGCTCTATGCTTATAACCTGTCTTT  GGAATCTTATCGATATCTGAATTAGTA |
| Forward and Reverse primer for *D. acutus* (QS primer) |
| GGCAATTCACTACACAGCCAACATCAAC  CCATAGTCAGGTGGTTAGTGATAC |
| **Universal Primer COI1490/ COI2198 (5’→3’)** |
| GGTCAACAAATCATAAAGATATTGG  TAAACTTCAGGGTGACCAAAAAATCA |

**Specific PCR amplification of *D. acutus*, *Z. dhumnades* and *B. multicinctus***

As demonstrated in Fig. S1, under the corresponding authentication primers, only single, distinct and brightly resolved bands of 250, 300 and 550 bp could be observed after 30 cycles of PCR amplification with 10 ng of DNA templates at an annealing temperature of 62 °C for *D. acutus, Z. dhumnades* and *B. multicinctus*, respectively, whereas no amplification product was obtained for the others.


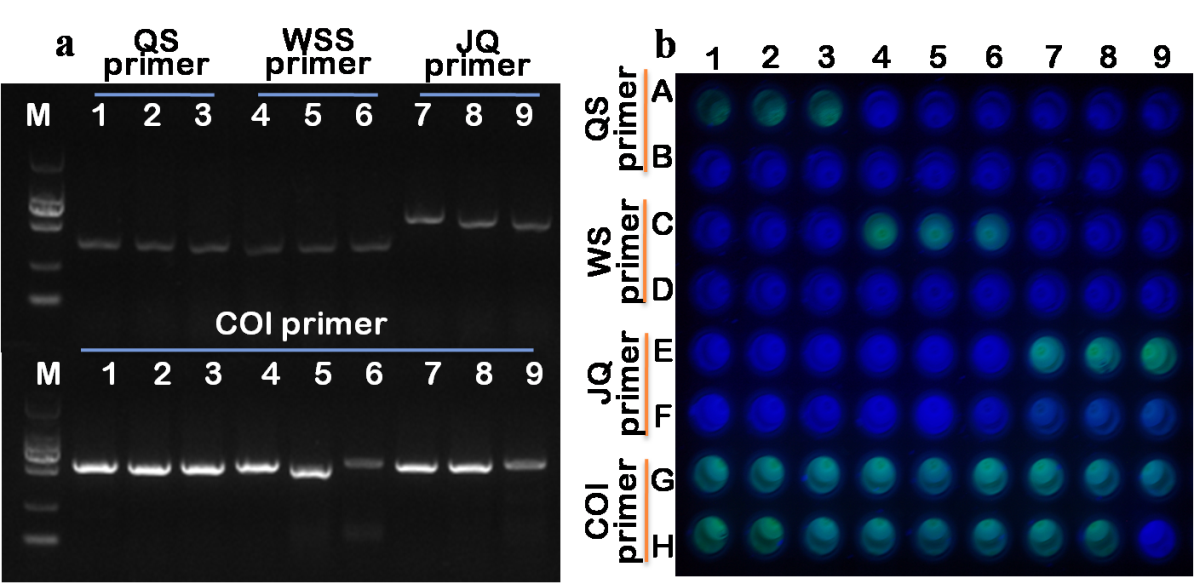


Fig. S1. (a) Specific PCR amplification results using the corresponding authentication primers. Electrophoresis was performed on a 2% agarose gel and stained with GelGreen. Band sizes of the DL 2000 DNA marker are indicated on the left. Lanes 1-3, 4-6 and 7-9 were *D. acutus*, *Z. dhumnades* and *B. multicinctus*, respectively. (b) Photograph of fluorescence patterns on a microplate corresponding to medicinal snakes and the adulterants. A1-3, C1-3, E1-3, and G1-3 were *D. acutus*; A4-6, C4-6, E4-6, and G4-6 were *Z. dhumnades*; A7-9, C7-9, E7-9, and G7-9 were *B. multicinctus*; B1-B8 were *B. fasciatus*, *Naja naja*, *Enhydris chinensis*, *Lycodon rufozonatus*, *Agkistrodon halys*, *Oocatochus rufodsata*, *Ptyas korros* and *Elaphe carinata*, respectively; D1-D8, F1-F8 and H1-H8 were identified with B1-B8; and B9, D9, F9 and H9 were no-template control (NTC) and used as the blank.

**References**

1 Duan, X. et al. Single-Nucleotide Polymorphism (SNP) Genotyping Using Cationic Conjugated Polymers in Homogeneous Solution. *Nat Protoc*. 4, 984-991 (2009).

2 Li, J., Wang, S., Yu, J., Wang, L. & Zhou, S. A Modified CTAB Protocol for Plant DNA Extraction. *Bullet. Bot*. 72-78 (2013).
